# Paclitaxel plus cetuximab for the treatment of R/M SCCHN after first-line pembrolizumab failure: primary analysis from the PaceAce trial

**DOI:** 10.1016/j.esmoop.2026.106061

**Published:** 2026-02-05

**Authors:** T. Fuereder, K. Klinghammer, D. Hahn, B. Grünberger, T. Melchardt, R. Greil, F. Kocher, G. Gamerith, C. Wagner, L. Berchtold, M. Burian, A. Strobl

**Affiliations:** 1 Department of Medicine I, Division of Oncology, Medical University of Vienna, Vienna, Austria; 2 Department of Hematology, Oncology and Cancer Immunology, Campus Benjamin Franklin, Charité—Universitätsmedizin Berlin, corporate member of Freie Universität Berlin and Humboldt-Universität zu Berlin, Berlin, Germany; 3 Department of Hematology, Oncology, Stem-Cell Transplantation and Palliative Care, Klinikum Stuttgart, Stuttgart, Germany; 4 Department of Medicine III, Hematology and Medical Oncology, Universitätsklinikum Wiener Neustadt, Wiener Neustadt, Austria; 5 III^rd^ Medical Department, Paracelsus Medical University, Cancer Center, Salzburg Cancer Research Institute, Cancer Cluster Salzburg, Salzburg, Austria; 6 Department of Internal Medicine V (Hematology and Oncology), Medical University of Innsbruck, Innsbruck, Austria; 7 Center for Medical Data Science, Institute of Medical Statistics, Medical University of Vienna, Vienna, Austria; 8 Department of ORL, Head and Neck Surgery, Ordensklinikum Linz, Barmherzige Schwestern, Linz, Austria

**Keywords:** head and neck cancer, paclitaxel, cetuximab, immunotherapy failure, recurrent/metastatic

## Abstract

**Background:**

No standard second-line treatment has been established for patients with recurrent or metastatic (R/M) squamous-cell carcinoma of the head and neck (SCCHN) progressing after first-line pembrolizumab-based therapy, representing a critical evidence gap in current clinical practice.

**Patients and methods:**

Patients with R/M SCCHN of the oropharynx, hypopharynx, larynx, or oral cavity, progressing after first-line pembrolizumab-based regimens, received paclitaxel (PTX) 175 mg/m^2^ every 21 days plus weekly cetuximab (C) 250 mg/m^2^ for up to six cycles, followed by C maintenance. The primary endpoint was overall response rate (ORR) at 12 weeks. Secondary endpoints included best overall response (BoR), disease control rate (DCR), progression-free survival (PFS), overall survival (OS), duration of response (DoR), quality of life, and safety.

**Results:**

Fifty-seven patients were enrolled (median age 64 years). Twenty-five patients (43.9%) had a primary tumor in the oropharynx, 17 (29.8%) in the oral cavity, 9 (15.8%) in the hypopharynx, and 6 (10.5%) in the larynx. The ORR was 43.9% [95% confidence interval (CI) 30.7% to 57.6%], the BoR was 47.4% (95% CI 34.0% to 61.0%) with nine (15.8%) complete responses, and the DCR was 71.9% (95% CI 58.5% to 83.0%). DoR was 5.7 months (95% CI 5.1 months-not reached). Median PFS and OS were 5.9 months (95% CI 5.5-8.4 months) and 12.2 months (95% CI 10.5-17.6 months), respectively. Six-month PFS and OS rates were 49.0% and 73.0%, respectively. The most frequent non-hematological treatment-related adverse events were C-associated skin rash (78.9%) and PTX-related polyneuropathy (35.1%).

**Conclusions:**

This is the first prospective trial specifically evaluating PTX plus C after failure of first-line pembrolizumab-based therapy in patients with R/M SCCHN. The observed clinical activity and tolerability support this widely available regimen as a potential standard-of-care option in the absence of randomized evidence in this setting.

## Highlights

• PaceAce is the first prospective trial testing PTX–C after first-line P failure in R/M SCCHN.
• PTX plus C achieved an ORR of 43.9% at 12 weeks and a DoR of 5.7 months in this setting.
• Median OS (12.2 months) and PFS (5.9 months) exceeded historical benchmarks from the pre-immunotherapy era.

## Introduction

Current international guidelines, including the National Comprehensive Cancer Network (NCCN) and European Society for Medical Oncology (ESMO) guidelines, provide limited guidance for patients with recurrent or metastatic squamous-cell carcinoma of the head and neck (R/M SCCHN) following progression on first-line immunotherapy.^1^^,^^2^ Recommendations for subsequent systemic treatment largely rely on evidence generated in the pre-immunotherapy era and do not account for prior checkpoint inhibitor exposure or declining performance status, which frequently precludes the use of platinum-based doublets in clinical practice. For example, the 2020 ESMO guidelines list platinum–5-fluorouracil (5-FU) plus cetuximab (C) as a potential second-line option only with a level IV recommendation, underscoring the lack of prospective data in this setting.^1^

The prognosis for patients with locoregionally unresectable R/M SCCHN remains poor. The advent of immune-checkpoint inhibitors, such as pembrolizumab (P), has revolutionized the treatment of R/M SCCHN. In the first-line setting, the phase III KEYNOTE-048 study defined a new standard of care (SoC). This study evaluated the efficacy of P monotherapy and pembrolizumab plus chemotherapy (P + chemo) versus the EXTREME regimen, and demonstrated an overall survival (OS) benefit for this approach, leading to the worldwide approval in R/M HNSCC patients with combined positive score (CPS) ≥1.^3^ Of note, comparable phase III studies with other immune-checkpoint inhibitors did not meet their endpoints and are not recommended.^4^^,^^5^

However, only a minority of patients respond to P therapy. The overall response rate (ORR) is ∼17% for P monotherapy and 36% for P + chemo in the total population.^3^

From a clinical perspective, the optimal management of patients who progress on or after SoC first-line P or P + chemo remains undefined and constitutes a major evidence gap.

On a molecular level, taxanes have been shown to interact with the immune system independently of their cytotoxic effects.^6^ Similarly, C modulates the immune response in addition to its activity against the epidermal growth factor receptor signaling pathway.^7^ In SCCHN, the contribution of immune-modulatory effects to the clinical response in C-treated patients has been well documented.^8^, ^9^, ^10^

While it is well known that both taxanes and C demonstrate clinical activity in the first-line setting as well as in the second-line setting after failure of conventional platinum-based therapy, their activity following P failure remains unclear and has not been studied in a prospective trial to date.^11^, ^12^, ^13^

Retrospective case series and one small prospective study (with heterogeneous histologies and prior treatments) have suggested activity of taxanes/C ± platinum after checkpoint inhibitors (CPI) given with or without chemotherapy, but the lack of consistency in design and patient populations precludes firm conclusions.^14^, ^15^, ^16^, ^17^, ^18^, ^19^, ^20^, ^21^, ^22^, ^23^, ^24^, ^25^

A recently published prospective study evaluating C monotherapy versus monalizumab plus C after failure of platinum and CPI therapy reported modest activity with an ORR of 23.9% in human papillomavirus-negative patients, highlighting the need for more effective regimens in this setting.^26^

We therefore conducted this prospective, international, multicenter phase II study to evaluate the efficacy and safety of paclitaxel (PTX) plus C in a homogeneous population of R/M SCCHN patients following first-line SoC P-based therapy.

## Patients and methods

### Study design and patients

This prospective, international, multicenter, single-arm phase II trial evaluated PTX plus C in patients with progressive disease after first-line P-based therapy for R/M SCCHN [≥6 months since last platinum exposure; all programmed death-ligand 1 (PD-L1) CPS ≥1 per approval status]. No interim therapies were permitted. The trial was conducted at seven centers in Austria and Germany from 10 February 2020 to 31 October 2024.

Eligible patients were aged ≥18 years, had an Eastern Cooperative Oncology Group (ECOG) performance status of ≤2, and had measurable R/M SCCHN of the oral cavity, oropharynx, larynx, or hypopharynx not amenable to surgery or radiotherapy. Full inclusion/exclusion criteria are provided in Supplementary Table S1, available at https://doi.org/10.1016/j.esmoop.2026.106061. On 19 March 2021, the protocol was amended to exclude patients treated with P in the platinum-resistant setting (<6 months since last platinum exposure). One such patient had been enrolled earlier and achieved a complete response (CR). After the amendment, another platinum-resistant patient was enrolled in deviation but excluded before baseline imaging due to non-compliance; data were retained in the database.

The study followed the Declaration of Helsinki and Good Clinical Practice guidelines and was approved by the ethics committee of the Medical University of Vienna (#2134/2019). The study is registered at ClinicalTrials.gov (NCT04278092).

### Procedures

All patients received C 400 mg/m^2^ loading dose intravenously followed by 250 mg/m^2^ weekly in combination with PTX 175 mg/m^2^ three weekly for up to six cycles followed by biweekly C maintenance 500 mg/m^2^ until tumor progression, unacceptable toxicity, or withdrawal of consent. While premedication with corticosteroids and antihistamines was recommended per protocol, supportive care—such as antibiotics for C-related skin rash or granulocyte colony-stimulating factor (G-CSF) support—was administered according to local guidelines at the investigators’ discretion.

### Study assessments and endpoints

The primary endpoint was ORR at 12 weeks, defined as the proportion of patients achieving partial response (PR) or CR to PTX plus C. Radiographic assessments [computed tomography (CT) or magnetic resonance imaging] were carried out at baseline and every 12 weeks until progression. Responses were investigator-assessed per RECIST 1.1. Patients without post-baseline tumor evaluation due to death, clinical progression, or toxicity were considered non-responders.

Patients lost to follow-up before first imaging were replaced by another eligible patient but retained in the database. Secondary endpoints included best overall response (BoR), disease control rate (DCR), OS, progression-free survival (PFS), duration of response (DoR), quality of life (QoL), and safety.

DoR was defined as the time from first CR/PR to progression or death.

OS was defined as the time from inclusion to death from any cause; patients alive or lost to follow-up were censored at the last known alive date. PFS was defined as the time from inclusion to progression or death; patients alive without progression were censored at the last tumor assessment. Patients starting a new therapy without progression were censored at their last valid assessment before that therapy. Adverse events (AEs) were graded per National Cancer Institute Common Terminology Criteria for Adverse Events (CTCAE) v5.0.

Health-related QoL was assessed using the European Organisation for Research and Treatment of Cancer (EORTC) Quality-of-Life Questionnaire Core 30 (QLQ-C30) and Quality-of-Life Questionnaire Head and Neck Module (QLQ-H&N35).

### Statistical analysis

PaceAce was a multicenter, single-arm phase II study evaluating ORR in patients with R/M SCCHN treated with C plus PTX after P-based first-line therapy. A one-sided exact binomial test (α = 0.025) provided 83.6% power to detect a difference between the null hypothesis (ORR π_0_ ≤ 0.10, based on CheckMate 141 and KEYNOTE-040)^27^^,^^28^ and the alternative (ORR π_a_ ≥ 0.25, per Saleh et al.)^29^ with a sample size of 50. The study was deemed positive if ≥10 patients achieved CR or PR. Sample size calculation used nQuery Advisor® v7.0 (Statistical Solutions Ltd, Cork, Ireland).

ORR was reported as absolute numbers and percentages with two-sided exact 95% confidence intervals (CIs), based on the full analysis set. Patients without post-baseline tumor assessments due to death, clinical progression, or toxicity were classified as non-responders. The BoR categories were described by frequencies and percentages. Individual patients’ responses over time are reported as case studies.

OS, PFS, and DoR were estimated via the Kaplan–Meier method; median follow-up was calculated using the reverse Kaplan–Meier method. All efficacy and safety analyses used the intention-to-treat population (all patients receiving one or more doses).

QoL questionnaires were collected at baseline and every 6 weeks, on the day of each treatment visit (PTX/C or C alone), and were scored as previously described.^30^ Statistical differences in QoL endpoints included the evaluation of mean changes from baseline during the study period. For each QoL scale, a mixed model was estimated with the QoL endpoint as the outcome, time as a fixed effect, and random intercept and slope. Mean changes from baseline during the study period were calculated from the model estimates with 95% CIs at timepoints of interest. The clinically meaningful difference, indicating a change that would be detectable by patients, was assumed to be a score difference of ≥10 points. Missing values due to treatment-related AEs were imputed in sensitivity analyses (lowest value observed before missing observation; alternatively, subtracting fixed amounts from value imputed under missing at random assumption). All other missing values were considered as missing at random. Statistical analysis was carried out using R version 4.4.0 (24 April 2024, R Foundation for Statistical Computing, Vienna, Austria).

## Results

### Patients

**Figure 1:**
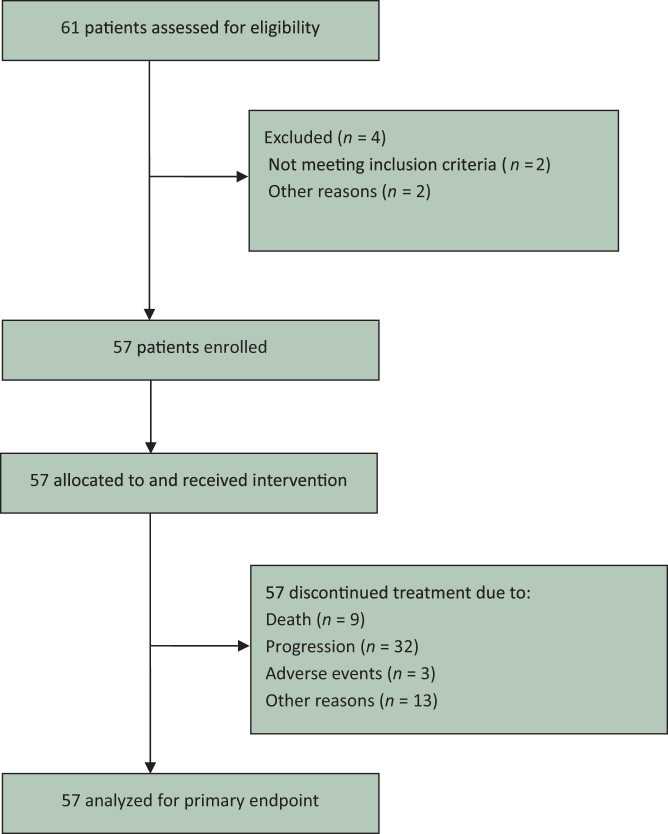
**CONSORT flow diagram of the study participants.** CONSORT, Consolidated Standards of Reporting Trials.

Fifty-seven patients with R/M SCCHN who had progressed on P-based first-line therapy were included in this study (Figure 1). Demographic and baseline clinical characteristics are summarized in Table 1. The patient cohort comprised 48 (84.2%) male and 9 (15.8%) female patients. The median age was 64 years (range 28-81 years), indicating a predominantly elderly population. While most patients had an ECOG performance status of 0-1, 7% had an ECOG status of 2.

**Table 1:** Patient and disease characteristics at baseline and treatment characteristics

Characteristics	Patients, *n* (%)
Sex	
Male	48 (84.2)
Female	9 (15.8)
Median age (range), years	64 (28-81)
Primary tumor site	
Oral cavity	17 (29.8)
Laryngeal carcinoma	6 (10.5)
Hypopharyngeal carcinoma	9 (15.8)
Oropharyngeal carcinoma	25 (43.9)
p16 status (oropharyngeal carcinoma)	
Positive	11 (44.0)
Negative	14 (56.0)
Disease status	
Recurrent	23 (40.4)
Metastatic	17 (29.8)
Recurrent and metastatic	17 (29.8)
ECOG status	
0	28 (49.1)
1	25 (43.9)
2	4 (7.0)
Previous first-line therapy	
Pembrolizumab monotherapy (P)	29 (50.9)
Pembrolizumab plus chemotherapy (P + chemo)	28 (49.1)
PD-L1 status	
CPS ≥1	55 (96.5%)
CPS 1-19	24 ( 42.1%)
CPS ≥20	31 (54.4%)
CPS unknown	2 (3.5%)

CPS, combined positive score; ECOG, Eastern Cooperative Oncology Group; PD-L1, programmed death-ligand 1.

Primary tumor sites included the oropharynx in 25 patients (43.9%), oral cavity in 17 patients (29.8%), hypopharynx in 9 patients (15.8%), and larynx in 6 patients (10.5%). Among oropharyngeal carcinoma cases, 44.0% were p16-positive. All patients had a PD-L1 CPS ≥1, as they were pre-treated with standard P-based first-line therapy per the European label. A CPS score of 1-19 was observed in 24 patients (43.6%), while 33 patients (56.4%) were high PD-L1 expressors (CPS ≥20).

Regarding disease status, 17 patients (29.8%) presented with metastatic disease, 23 (40.4%) had locoregional recurrence only, and 17 (29.8%) exhibited both recurrent and distant metastatic disease.

The median number of chemotherapy administrations with PTX was 6 (range 1-6), and the median number of C administrations was 19 (range 0-106).

### Efficacy

**Figure 2:**
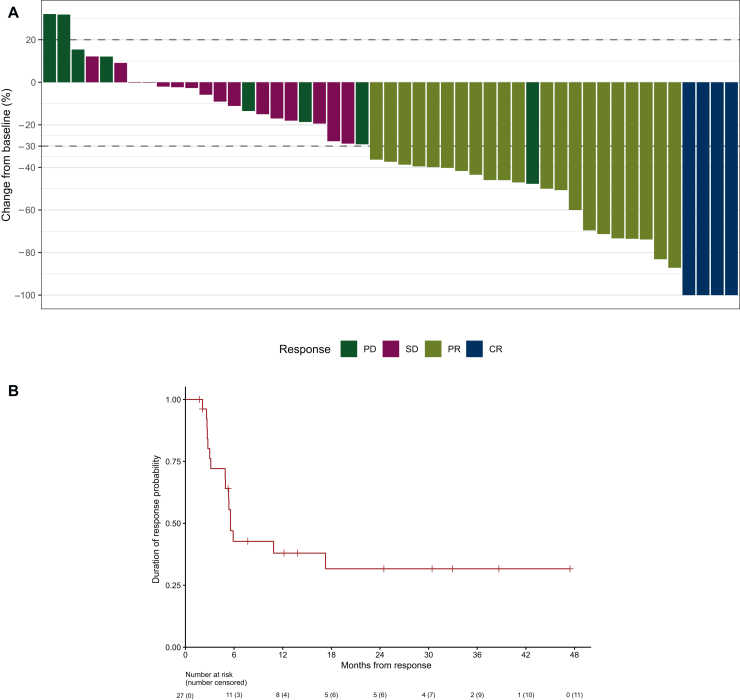
**Tumor response and duration of response.** Waterfall plot illustrating individual patients’ responses at 12 weeks. Complete response (CR), partial response (PR), stable disease (SD), and progressive disease (PD) (A) and Kaplan–Meier curves depicting median duration of response (DoR) (B).

**Figure 3:**
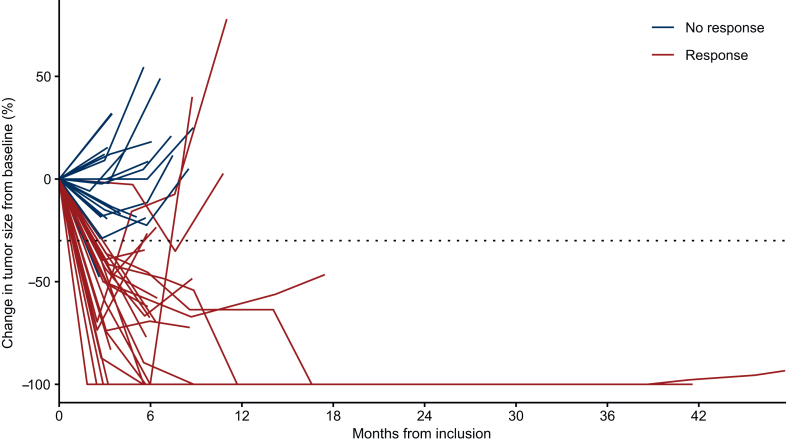
**Time-course of individual tumor burden.** Spider plot illustrating the longitudinal change in tumor size from baseline (%) over time.

The median follow-up time was 31.7 months [95% CI 27.4 months-not reached (NR)]. At 12 weeks, the primary endpoint analysis showed that a total of 23 patients achieved a response (CR or PR), resulting in an ORR of 43.9% (95% CI 30.7% to 57.6%) (Table 2, Figure 2A). This ORR was significantly higher than the predefined reference value of 10% (*P* < 0.001, one-sided exact test). Patients with p16-unrelated disease had an ORR of 41.3% (95% CI 27.0% to 56.8%) (Table 2). Over the entire treatment duration, 9 patients (15.8%) achieved CRs, and 18 patients (31.6%) had PRs, resulting in a BoR of 47.4% (95% CI 34.0% to 61.0%) (Supplementary Table S2 and Figure S1, available at https://doi.org/10.1016/j.esmoop.2026.106061). The median DoR was 5.7 months (95% CI 5.1 months-NR) (Figure 2B). Disease control was achieved in 41 patients, corresponding to a DCR of 71.9% (95% CI 58.5% to 83.0%) (Table 2). Individual tumor size changes are illustrated in Figure 3 as a spider plot.

**Table 2:** Summary of treatment results at 12 weeks

ORR at 12 weeks	Patients, *n* (*n* = 57)	%
CR	4	7.0
95% CI		0-21.3
PR	21	36.8
95% CI		24.6-51.2
SD	16	28.1
95% CI		15.8-42.4
Progressive disease	13	22.8
95% CI		10.5-37.1
Not evaluable	3	5.3
ORR (CR + PR)	25	43.9
95% CI		30.7-57.6
ORR (CR + PR) in p16-negative patients	19	41.3
95% CI		27.0-56.7
Disease control rate (CR + PR + SD)	41	71.9
95% CI		58.5-83.0

CI, confidence interval; CR, complete response; ORR, overall response rate; PR, partial response; SD, stable disease.

**Figure 4:**
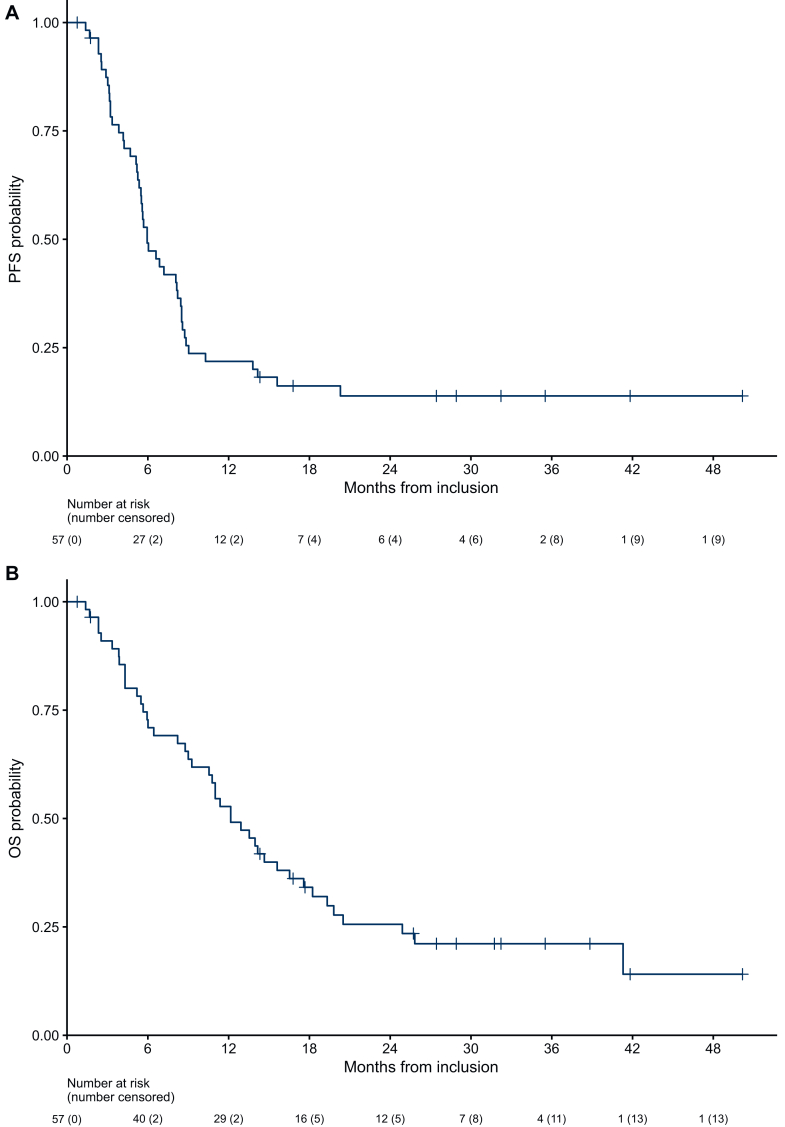
**Survival outcomes in the study population.** Kaplan–Meier curves depicting progression-free survival (PFS) (A) and overall survival (OS) (B).

The median PFS was 5.9 months (95% CI 5.5-8.4 months), and the median OS was 12.2 months (95% CI 10.5-17.6 months) (Figure 4). The 6-month and 12-month PFS rates were 49% and 22%, respectively, while the 6-month and 12-month OS rates were 73% and 53%, respectively.

**Figure 5:**
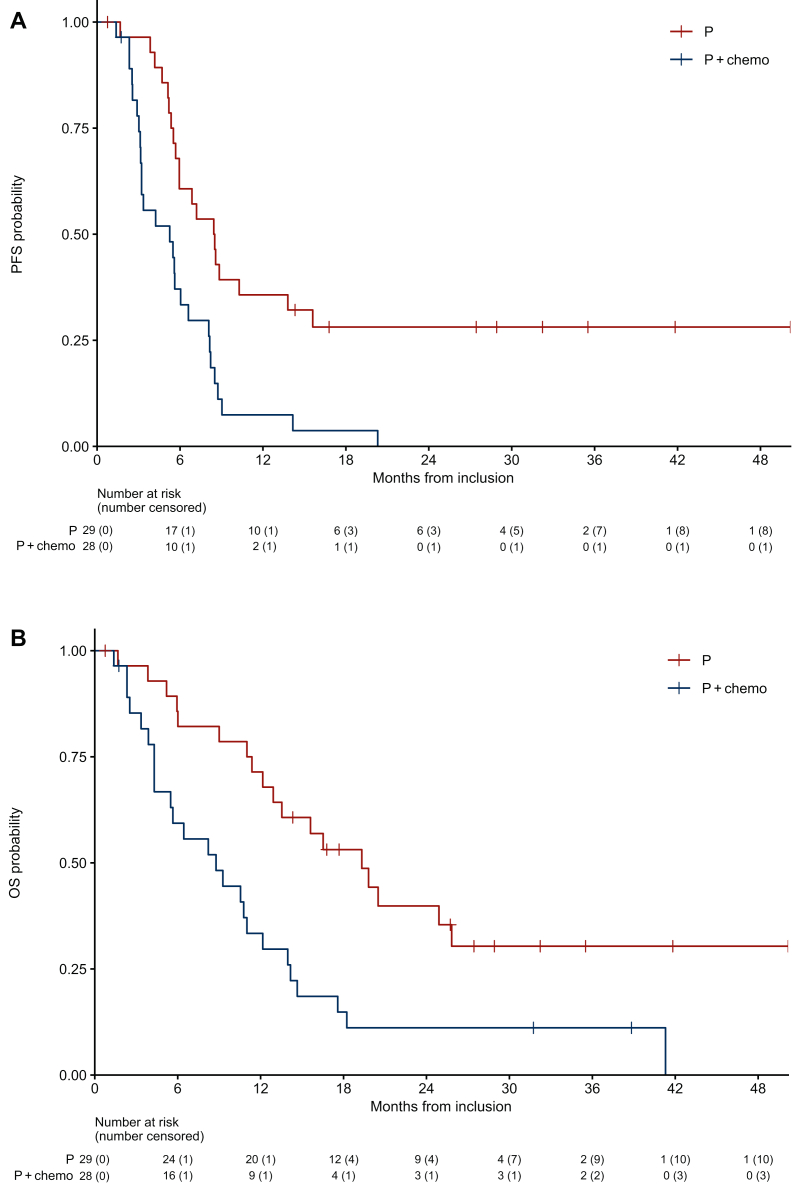
**Survival outcomes according to prior therapy.** Kaplan–Meier curves depicting progression-free survival (PFS) (A) and overall survival (OS) (B) of patients pre-treated with pembrolizumab (P) monotherapy or pembrolizumab plus chemotherapy (P + chemo).

An exploratory *post hoc* analysis showed that patients pre-treated with P monotherapy had a numerically higher BoR of 62.1% (95% CI 42.3% to 79.3%) and a longer median PFS of 8.5 months (95% CI 5.9 months-NR) compared with a BoR of 32.1% (95% CI 15.9% to 52.4%) and a PFS of 5.3 months (95% CI 3.2-8.1 months) in those pre-treated with P + chemo. Similarly, the median OS was 19.3 months (95% CI 12.9 months-NR) in the monotherapy group versus 8.8 months (95% CI 5.5-14 months) in the combination therapy group (Figure 5).

An additional exploratory OS analysis, calculated from the start of first-line P (± chemotherapy), showed a median OS of 18.8 months (95% CI 16.3-24.8 months) for the total population. In the subgroup of patients pre-treated with P monotherapy, the median OS was 25.3 months (95% CI 18.3 months-NR), whereas in patients pre-treated with P + chemo, the median OS was 16.7 months (95% CI 13.3-18.9 months) (Supplementary Figure S2, available at https://doi.org/10.1016/j.esmoop.2026.106061). The individual treatment course per therapy line is depicted in Supplementary Figure S2C, available at https://doi.org/10.1016/j.esmoop.2026.106061.

### Safety

The most frequent non-hematological treatment-related AE was C-associated skin rash, reported in 45 patients (78.9%), including 8 patients (14%) who experienced a grade ≥3 rash. The most common PTX-related AE was polyneuropathy, which occurred in 20 patients (35.1%). Grade ≥3 neutropenia was observed in 15 patients (26.3%), and febrile neutropenia was reported in 2 patients (3.5%). Notably, sepsis occurred in six patients (10.5%), with two of these cases resulting in death; these fatalities were deemed unrelated to the therapies by the investigators. Treatment-related grade 5 AEs included one case (1.8%) of ileus, attributed to PTX therapy. AEs are summarized in Supplementary Table S3, available at https://doi.org/10.1016/j.esmoop.2026.106061. Overall, the combination of PTX and C was well tolerated, with no new safety concerns identified.

### Quality of life

The number of QoL questionnaires returned was 44 at cycle 4, when the first re-staging scan was scheduled.

For the EORTC QLQ-C30 questionnaire, no clinically relevant decline by 10 points in the global health status, functional, and symptom scales was observed between baseline and cycle 4 at the first imaging. However, a decline in physical functioning over time was observed, with a mean change of −9.9 points (95% CI −16.48 to −3.37 points, *P* = 0.003) between baseline and first re-staging and a change of −13.13 points (95% CI −20.13 to −6.14 points, *P* < 0.001) between baseline and end of treatment (EOT) (Supplementary Table S4, available at https://doi.org/10.1016/j.esmoop.2026.106061).

Regarding the EORTC H&N35 scale, patients primarily reported a deterioration in coughing, with a mean change of −18.49 points (95% CI −16.48 to −3.37 points, *P* = 0.003) as well as difficulties with mouth opening. Patients also required more analgesics at the first imaging (CT) and EOT compared with baseline as shown in a logistic model [CT: odds ratio (OR) 0.053, 95% CI 0.01-0.294, *P* < 0.001; EOT: OR 0.144, 95% CI 0.03-0.685, *P* = 0.015] (Supplementary Table S5, available at https://doi.org/10.1016/j.esmoop.2026.106061). The sensitivity analysis, conducted per protocol, imputed missing QoL scores due to treatment-related AEs using the lowest observed pre-missing value, while values missing due to progression were not imputed. Results remained consistent with the primary analysis, confirming deterioration in coughing at CT and EOT. For other scales, such as painkiller use and physical functioning, no imputation was necessary.

## Discussion

The PaceAce study is the first prospective trial to address the post-immunotherapy evidence gap in a typical second-line R/M SCCHN population including patients with an ECOG performance status of 0-2.

While retrospective studies after CPI failure have reported heterogeneous outcomes with ORRs ranging from 22% to 60% and median OS between 6 and 23 months,^15^, ^16^, ^17^, ^18^, ^19^, ^20^, ^21^, ^22^, ^23^, ^24^, ^25^^,^^29^ their interpretability is limited by differences in patient populations, regimens, and CPI used.

In contrast, trials such as CheckMate 141 and KEYNOTE-040 reported substantially lower response rates (ORR 10%-13%, OS 5-7 months) in the SoC arms, which consisted of chemotherapy or C.^27^^,^^28^

One prospective study by Koyama et al. evaluated weekly PTX plus biweekly C after platinum and CPI therapy and reported a high ORR of 69.6%.^14^ However, key differences—heterogeneous tumor sites, performance status, prior C exposure, predominance of nivolumab pre-treated patients, and lack of PD-L1 data—limit comparability with PaceAce, which enrolled exclusively R/M SCCHN patients post-P with CPS ≥1 and excluded prior C. Despite these differences, survival outcomes were comparable (PFS 5.9 versus 5.5 months; OS 12.2 versus 13.3 months), and both exceeded historical benchmarks.

In an exploratory analysis, outcomes were more favorable in patients previously treated with P monotherapy than in those who had received P + chemo. This likely reflects differences in baseline disease burden and biology rather than differential efficacy of PTX + C. Specifically, P monotherapy is often reserved for patients with lower burden and higher CPS, whereas P + chemo is used in more aggressive disease, potentially confounding outcome interpretation.

Of note, prior platinum exposure in the P + chemo pre-treated subgroup deserves additional attention: while it is not surprising that this might have led to inferior outcomes with PTX plus C compared with the P monotherapy subgroup, it has to be stated that trials of taxanes plus C conducted in the pre-immunotherapy era in platinum pre-treated patients resulted in an ORR of 11%, a PFS of 3.1 months, and an OS of 6.7 months, which is lower than what we observed.^13^

While it remains unclear whether treatment intensification to a platinum doublet plus C regimen might have resulted in better outcomes in patients who progressed on P monotherapy compared with P + chemo, it is noteworthy that the BoR of 62.1% and the OS of 19.3 months observed with PTX plus C in this subgroup are similar to those reported in the first-line setting with platinum/taxane plus C regimens, such as in the TPEX study.^31^

No new safety signals were detected with PTX plus C and the most frequent AEs included C-associated skin rash and PTX-related polyneuropathy. Although primary prophylaxis with G-CSF was not mandated by protocol, the incidence of sepsis (10.5%), including two fatal cases, highlights the importance of individualized risk assessment. Despite a low rate of febrile neutropenia (3.5%), the spectrum and severity of infections observed suggest that prophylactic use of G-CSF should be considered more broadly in this setting, particularly in patients with predisposing risk factors.

QoL is an independent prognostic indicator of OS in R/M SCCHN.^32^, ^33^, ^34^ In the PaceAce study, we did not observe a major decline in the global health status of patients. However, declines in physical functioning, deterioration in mouth opening, and an increased need for analgesics were observed, reflecting tumor progression over time.

The PaceAce trial has several limitations. (i) Its single-arm design limits the ability to draw definitive conclusions about relative efficacy and prognostic subgroups deriving additional benefit from this approach. However, setting up a randomized study in this context may be challenging, as no SoC option has been established to serve as a comparator arm for PTX plus C. (ii) The study design intentionally moved away from the traditional distinction between platinum-resistant and platinum-sensitive patients, focusing instead on P-resistant disease. This reflects the current practice and acknowledges that durable responses to P maintenance after platinum-based chemoimmunotherapy are common. However, this approach resulted in a patient population in PaceAce that was both platinum-sensitive (i.e. P monotherapy pre-treated) and platinum-resistant (i.e. P plus platinum/5-FU pre-treated). Given that the null hypothesis was derived from studies in platinum-refractory patients, it may underestimate the expected ORR in a platinum-sensitive, P monotherapy-resistant population, which may not fully align with the mixed platinum sensitivity of the PaceAce cohort.

Moreover, platinum-based chemotherapy regimens, such as EXTREME or TPEX, are not feasible for all patients in the second-line setting. In daily practice, a significant proportion of patients progressing after first-line P therapy present with declining performance status or cumulative toxicities that limit the use of platinum doublets.

Notably, the PaceAce trial included patients with an ECOG performance status of 2 (7%), who would likely not have been eligible for platinum-based salvage therapy. This underlines the clinical relevance and broader applicability of PTX plus C in this setting.

### Conclusion

The PaceAce study is the first prospective trial specifically evaluating PTX plus C after failure of first-line P-based therapy in patients with R/M SCCHN. These results address a critical post-immunotherapy evidence gap and provide prospective data supporting the use of this combination as a widely accessible second-line treatment option. Notably, the inclusion of patients with an ECOG performance status of 2 enhances the generalizability of the findings to routine clinical practice, where platinum-based doublets are often no longer feasible. In the absence of a randomized controlled trial, these data substantiate current NCCN and ESMO guidelines and offer a rational, evidence-informed approach to SoC second-line management in this population.
