# Supplementary material for: Paclitaxel plus cetuximab for the treatment of R/M SCCHN after first-line pembrolizumab failure: primary analysis from the PaceAce trial
Source: ESMO Open. 2026 Feb 5;11(2):106061. doi: 10.1016/j.esmoop.2026.106061 (PMC12906205; doi:10.1016/j.esmoop.2026.106061)
Supplement: Supplementary Material [file mmc1.docx]

| Inclusion Criteria |
| --- |
| - The patient has provided written informed consent prior to any study-related procedure. |
| - The patient is at least 18 years of age |
| - Histologically proven locally advanced unresectable, recurrent and/or metastatic squamous cell carcinoma of the oropharynx, hypopharynx, larynx or oral cavity not amenable for salvage surgery |
| - p16 status has to be determined for oropharyngeal carcinomas |
| - Documented progressive disease based on investigator assessment according to RECIST 1.1, following receipt of a pembrolizumab based regimen given as first line therapy in the platinum sensitive setting (i.e. ≥ 6 months since last platinum exposure) for recurrent and/or metastatic SCCHN |
| - Measurable disease according to RECIST 1.1. |
| - The patient has a life expectancy of at least 3 months. |
| - Has a performance of ≤ 2 on the ECOG Performance Scale |
| - Female patient of childbearing potential should have a negative urine or serum pregnancy 24 hours prior to treatment initiation. If the urine test is positive or cannot be confirmed as negative, a serum pregnancy test will be required. |
| - Female patients of childbearing potential must agree to use highly effective contraception (refer to section 10) or be surgically sterile, or abstain from heterosexual activity for the course of the study until 120 days after the last dose of study medication. Patients of childbearing potential are those who have not been surgically sterilized or have not been free from menses for > 1 year. |
| - Male patients must agree to use an adequate method of contraception (condom) starting with the first dose of study therapy until 120 days after the last dose of study therapy (refer to section 10). |
| - Demonstrate adequate organ function, all screening labs should be performed within 14 days of treatment initiation. |
|  |
| Exclusion Criteria |
| - Prior taxane therapy is not allowed except as part of induction therapy for locally advanced disease (completed at least 6 months before study entry) |
| - Prior cetuximab therapy is not allowed except as part of either induction therapy or in combination with radiotherapy treatment for locally advanced disease (completed at least 6 months before study entry) |
| - Patients with nasopharyngeal carcinomas or salivary glands cancers |
| - Is currently participating and receiving study therapy or has participated in a study of an investigational agent and received study therapy within 4 weeks of the first dose of treatment. |
| - Has a diagnosis of immunodeficiency including a known history of Human Immunodeficiency Virus (HIV) (HIV 1/2 antibodies). |
| - Has known active Hepatitis A/B or Hepatitis C |
| - Has had prior pembrolizumab within 1 week prior to study day 1 or who has not recovered (i.e., recovery to ≤ Grade 1 or baseline grade prior to pembrolizumab) from (immune- related) adverse events other than endocrine side effects. |
| - Has had prior chemotherapy or radiation therapy within 2 weeks prior to study day 1 or who has not recovered (i.e., recovery to ≤ Grade 1 or baseline grade prior to pembrolizumab) from adverse events due to a previously administered agent. |
| - Has had chemotherapy, targeted therapy or investigational drugs after checkpoint inhibitor failure for second line therapy. |
| - Has had prior pembrolizumab in the platinum resistant setting (<6 months after last platinum exposure). |
| - Has a known additional malignancy that is progressing or requires active treatment. Exceptions include basal cell carcinoma of the skin or squamous cell carcinoma of the skin that has undergone potentially curative therapy or in situ cervical cancer. |
| - Has an active infection requiring systemic therapy. |
| - Has a history or current evidence of any condition, therapy, or laboratory abnormality that might confound the results of the trial, interfere with the patient’s participation for the full duration of the trial, or is not in the best interest of the patient to participate, in the opinion of the treating investigator. |
| - Has known psychiatric or substance abuse disorders that would interfere with cooperation with the requirements of the trial. |
| - Is pregnant or breastfeeding, or expecting to conceive or father children within the projected duration of the trial, starting with the pre-screening or screening visit until 120 days after the last dose of trial treatment. |
| - Any direct relationship to the sponsor, the investigator or the trial site |
| - Placement in an institution on the basis of a judicial or administrative order |

**Supplementary table 1**: Eligibility criteria.

| **Best Response** | **Number of Patients (n=57)** | **Percentage** |
| --- | --- | --- |
|  |  |  |
| **CR** | **9** | **15.8%** |
| **95% CI** |  | **3.5% to 29.8%** |
| **PR** | **18** | **31.6%** |
| **95% CI** |  | **19.3% to 45.6%** |
| **Stable Disease** | **14** | **24.6%** |
| **95% CI** |  | **12.3% to 38.5%** |
| **Progressive Disease** | **13** | **22.8%** |
| **95% CI** |  | **10.5% to 36.8%** |
| **Not evaluable** | **3** | **5.3%** |
|  |  |  |
| **Best Overall Response Rate (CR+PR)** | **27** | **47.4%** |
| **95% CI** |  | **34.0% to 61.0%** |
| **Best Overall Response Rate (CR+PR)** | **20** | **43.5%** |
| **in p16 neg. patients** |  |  |
| **95% CI** |  | **28.9% to 58.9%** |

**Supplementary table 2**: Best overall response rate (BoR).

| **Adverse event** | **Grade 1-2** | **Grade 3-5** |
| --- | --- | --- |
| **Blood and lymphatic system disorders**  Anemia  Neutropenia and leukopenia  Leukocytosis  Febrile Neutropenia  Thrombocytopenia | 7 (12.3%)  5 (8.8%)  1(1.8%)  5 (8.8%) | 4 (7.0%)  15 (26.3%)  1 (1.8%)  2 (3.5%)  1 (1.8%) |
| **Cardiac Disorders**  Chest pain  Myocardial Infarction | 1 (1.8%) | 1 (1.8%) |
| **Endocrine disorders**  Hypothyroidism | 1 (1.8%) |  |
| **Eye Disorders**  Cataract  Keratitis | 1(1.8%)  2 (3.5%) |  |
| **Gastrointestinal disorders**  Anal fissure  Constipation  Dark stool  Diarrhea  Duodenitis  Dry Lip  Dysphagia  Gastroparesis  Gastroesophageal reflux disease  Hematochezia  Ileus and Ischemia  Nausea  Mucositis and Ulceration  Oral Dysesthesia | 1 (1.8%)  7 (12.3%)  2 (3.5%)  8 (14.0%)  1 (1.8%)  1 (1.8%)  6 (10.5%)  3 (5.3%)  1 (1.8%)  10 (17.5%)  10 (17.5%)  3 (5.3%) | 1 (1.8%)  2 (3.5%)  2 (3.5%)  2 (3.5%) |
| **General disorders**  Chills  Edema  Fatigue  General condition worsening  Pyrexia  Extravasation  Sudden death | 2 (3.5%)  8 (14.0%)  11  5 (8.8%)  4 (7.0%) | 2 (3.5%)  3 (5.3%)  2 (3.5%) |
| **Immune System Disorders**  Allergic Reaction | 1 (1.8%) | 3 (5.3%) |
| **Metabolism and nutrition disorders**  Anorexia  Dyslipidemia  Electrolyte Deficiency  Folate-deficiency  Hypercalcemia  Hypocalcemia  Hyperkalemia  Hypokalemia  Hypomagnesaemia  Hypophosphatemia  Hypoglycemia | 3 (5.3%)  1 (1.8%)  1 (1.8%)  1 (1.8%)  1(1.8%)  3 (5.3%)  5 (8.8%)  7 (12.3%)  1 (1.8%)  1 (1.8%) | 1 (1.8%)  1 (1.8%)  1(1.8%)  2 (3.5%) |
| **Renal and urinary disorders**  Hematuria  Urinary tract obstruction (Hydronephrosis) | 1 (1.8%) | 1 (1.8%) |
| **Respiratory, thoracic, mediastinal disorders**  Cough  Dyspnea  Emphysema  Epistaxis  Hemoptysis  Hiccups  Hoarseness | 7 (12.3%)  2 (3.5%)  1 (1.8%)  1 (1.8%)  1 (1.8%)  1 (1.8%)  1 (1.8%) | 1 (1.8%) |
| **Reproductive system and breast disorders**  Gynecomastia | 1 (1.8%) |  |
| **Skin and subcutaneous disorders**  Alopecia  Dermatitis  Fistula  Hyperkeratosis  Nail discoloration  Rash  Rhagades  Pruritus  Skin ulceration  Skin alteration including traumatic | 9 (15.8%)  5 (8.8%)  2 (3.5%)  1 (1.8%)  1 (1.8%)  37 (64.9%)  3 (5.3%)  4 (7.0%)  1 (1.8%)  7 (12.3%) | 8 (14.0%) |
| **Neoplasms malignant and unspecified**  Fungating wound  Tumor hemorrhage  Mouth opening disturbed  Mucus, sticky or increased saliva  Pain | 2 (3.5%)  3 (5.3%)  1 (1.8%)  3 (5.3%)  1 (1.8%) | 2 (3.5%)  1 (1.8%) |
| **Musculoskeletal and connective tissue**  Arthritis  Burstitis  Muscle cramp and stiffness  Generalized muscle weakness  Pain (non-tumor)  Vertebral Dislocation | 1 (1.8%)  3 (5.3%)  9 (15.8%)  14 (24.6%) | 1 (1.8%) |
| **Investigations**  Creatinine increased  Liver enzymes increased  CRP increased  Pancreatic enzymes increased  PSA elevation  Weight loss | 1 (1.8%)  6 (10.5%)  5 (8.8%)  2 (3.5%)  1 (1.8%)  5 (8.8%) | 2 (3.5%)  1 (1.8%)  1 (1.8%) |
| **Ear and labyrinth disorders**  Vertigo  Otic polyp  Tinnitus | 7 (12.3%)  2 (3.5%)  1 (1.8%) | 1 (1.8%) |
| **Nervous system disorders**  Dysgeusia  Headache  Neuralgia after Herpes  Peripheral sensory neuropathy  Peroneal palsy  Stroke and TIA  Syncope, Absence and Collapse  Amnesia | 2 (3.5%)  5 (8.8.%)  1 (1.8%)  20 (35.1%)  2 (3.5%)  3 (5.3%)  1 (1.8%) | 1 (1.8%)  2 (3.5%)  2 (3.5%)  4 (7.0%) |
| **Psychiatric disorders**  Anxiety  Depression  Suicide attempt | 1 (1.8%) | 2 (3.5%)  1 (1.8%) |
| **Infections and infestations**  Balanitis  Bursitis  Conjuctivitis  COVID infection  Herpes infection  Upper respiratory infection  Infection unspecified  Paronychia  Pneumonia  Port infection  Skin Infection (bacterial and fungal)  Sepsis  Urinary Tract Infection  Wound infection | 2 (3.5%)  1 (1.8%)  3 (5.3%)  5 (8.8%)  2 (3.5%)  1 (1.8%)  10 (17.5%)  2 (3.5%)  3 (5.3%)  3 (5.3%)  2 (3.5%)  3 (5.3%) | 2 (3.5%)  6 (10.5%)  1 (1.8%)  1 (1.8%)  6 (10.5%)  2 (3.5%) |
| **Injury, poisoning and procedural complications**  Tracheostomy and PEG tube bleeding  Fracture  Port complications  Wound complication including healing disorders | 3 (5.3%)  1 (1.8%) | 2 (3.5%)  3 (5.3%)  1 (1.8%) |
| **Vascular Disorders**  Chronic venous insufficiency  Hypertension  Thrombus  Vasculitis | 1 (1.8%)  1 (1.8%)  2 (3.5%)  1 (1.8%) |  |

**Supplementary table 3**: Adverse events graded by CTCAE 5.0.

| Outcome | Term | Estimate Coeffient | 95% CI | p-value |
| --- | --- | --- | --- | --- |
| QL2 | (Intercept) | 47.978 | 42.081 - 53.874 | <0.001 |
|  | BL – week 12 | 7.191 | 0.413 - 13.968 | 0.038 |
|  | BL - EOT | -1.702 | -8.914 - 5.509 | 0.64 |
| PF2 | (Intercept) | 67.143 | 59.97 - 74.316 | <0.001 |
|  | BL - week 12 | -9.926 | -16.478 - -3.374 | 0.003 |
|  | BL - EOT | -13.133 | -20.125 - -6.141 | <0.001 |
| RF2 | (Intercept) | 51.190 | 41.607 - 60.774 | <0.001 |
|  | BL - week 12 | -0.193 | -10.062 - 9.675 | 0.969 |
|  | BL - EOT | -9.717 | -20.235 - 0.801 | 0.07 |
| EF | (Intercept) | 60.670 | 53.122 - 68.218 | <0.001 |
|  | BL - week 12 | 7.570 | 0.169 - 14.972 | 0.045 |
|  | BL - EOT | 1.051 | -6.841 - 8.944 | 0.792 |
| CF | (Intercept) | 79.663 | 72.688 - 86.638 | <0.001 |
|  | BL - week 12 | 0.673 | -5.396 - 6.743 | 0.826 |
|  | BL - EOT | -5.451 | -11.93 - 1.029 | 0.098 |
| SF | (Intercept) | 58.659 | 49.411 - 67.907 | <0.001 |
|  | BL - week 12 | 1.069 | -7.724 - 9.862 | 0.809 |
|  | BL - EOT | 6.587 | -2.849 - 16.024 | 0.169 |
| FA - rev | (Intercept) | 50.099 | 42.013 - 58.185 | <0.001 |
|  | BL - week 12 | -2.524 | -10.436 - 5.388 | 0.528 |
|  | BL - EOT | -6.628 | -15.066 - 1.81 | 0.122 |
| NV - rev | (Intercept) | 88.988 | 83.444 - 94.533 | <0.001 |
|  | BL - week 12 | -3.392 | -9.696 - 2.911 | 0.288 |
|  | BL - EOT | 1.960 | -4.748 - 8.668 | 0.563 |
| PA - rev | (Intercept) | 55.357 | 46.945 - 63.769 | <0.001 |
|  | BL - week 12 | 8.385 | -0.682 - 17.452 | 0.069 |
|  | BL - EOT | -0.123 | -9.78 - 9.534 | 0.98 |

**Supplementary Table 4**: Summay: Linear Mixed Models for each EORTC QLQ-C30 score by timepoints of interest (baseline (BL), at first CT assessment scheduled at week 12 (median time to assessment 13.0 weeks) and end of treatment (EOT). The timepoints are used as fixed effects and random intercept for each patient.

| Linear Models | | | | |
| --- | --- | --- | --- | --- |
| Outcome | Term | Estimate Coeffient | 95% CI | p-value |
| Pain | (Intercept) | 24.058 | 18.15 - 29.965 | <0.001 |
|  | BL - week 12 | -3.934 | -8.869 - 1 | 0.122 |
|  | BL - EOT | -3.946 | -9.271 - 1.379 | 0.15 |
| Swallowing | (Intercept) | 45.356 | 36.367 - 54.345 | <0.001 |
|  | BL - week 12 | -8.086 | -13.575 - -2.597 | 0.005 |
|  | BL - EOT | -6.525 | -12.428 - -0.622 | 0.033 |
| Senses problems | (Intercept) | 30.606 | 22.763 - 38.449 | <0.001 |
|  | BL - week 12 | -6.090 | -13.032 - 0.852 | 0.089 |
|  | BL - EOT | -6.080 | -13.503 - 1.342 | 0.112 |
| Speech problems | (Intercept) | 35.168 | 26.362 - 43.973 | <0.001 |
|  | BL - week 12 | 4.063 | -4.012 - 12.138 | 0.327 |
|  | BL - EOT | 7.633 | -1.014 - 16.28 | 0.087 |
| Trouble with social eating | (Intercept) | 47.753 | 38.134 - 57.371 | <0.001 |
|  | BL - week 12 | -9.202 | -17.266 - -1.138 | 0.028 |
|  | BL - EOT | -1.389 | -9.882 - 7.104 | 0.749 |
| Trouble with social contact | (Intercept) | 21.633 | 14.913 - 28.352 | <0.001 |
|  | BL - week 12 | 0.802 | -4.61 - 6.214 | 0.772 |
|  | BL - EOT | 1.622 | -4.224 - 7.467 | 0.588 |
| Less sexuality | (Intercept) | 44.806 | 34.377 - 55.235 | <0.001 |
|  | BL - week 12 | 2.536 | -7.385 - 12.458 | 0.618 |
|  | BL - EOT | 9.076 | -1.707 - 19.858 | 0.103 |
| Teeth | (Intercept) | 27.710 | 18.024 - 37.396 | <0.001 |
|  | BL - week 12 | -9.714 | -19.897 - 0.47 | 0.065 |
|  | BL - EOT | -5.903 | -16.743 - 4.936 | 0.289 |
| Opening mouth | (Intercept) | 45.743 | 34.709 - 56.776 | <0.001 |
|  | BL - week 12 | -10.287 | -19.698 - -0.876 | 0.035 |
|  | BL - EOT | 2.263 | -7.789 - 12.316 | 0.66 |
| Dry mouth | (Intercept) | 36.970 | 27.803 - 46.136 | <0.001 |
|  | BL - week 12 | -2.634 | -11.456 - 6.189 | 0.56 |
|  | BL - EOT | -0.095 | -9.442 - 9.252 | 0.984 |
| Sticky saliva | (Intercept) | 53.543 | 43.562 - 63.523 | <0.001 |
|  | BL - week 12 | -3.706 | -12.36 - 4.949 | 0.404 |
|  | BL - EOT | -5.319 | -14.584 - 3.946 | 0.264 |
| Coughing | (Intercept) | 47.024 | 38.316 - 55.731 | <0.001 |
|  | BL - week 12 | -18.490 | -27.686 - -9.293 | <0.001 |
|  | BL - EOT | -12.336 | -22.237 - -2.435 | 0.017 |
| Felt ill | (Intercept) | 45.238 | 35.53 - 54.946 | <0.001 |
|  | BL - week 12 | -4.255 | -15.34 - 6.829 | 0.454 |
|  | BL - EOT | 1.558 | -10.359 - 13.475 | 0.798 |
|  | | | | |
| Logistic Model | | | | |
| Outcome | Term | Odds Ratio (OR) | 95% CI | p-value |
| Pain killers | (Intercept) | 17.322 | 2.697 - 111.274 | 0.003 |
|  | BL - week 12 | 0.053 | 0.010 - 0.294 | <0.001 |
|  | BL - EOT | 0.144 | 0.030 - 0.685 | 0.015 |
| Nutritional supplements | (Intercept) | 0.596 | 0.288 - 1.232 | 0.163 |
|  | BL - week 12 | 0.540 | 0.201 - 1.451 | 0.222 |
|  | BL - EOT | 0.478 | 0.164 - 1.467 | 0.177 |
| Feeding tube | (Intercept) | 0.000 | 0.000 - 0.006 | <0.001 |
|  | BL - week 12 | 1.657 | 0.228 - 12.049 | 0.618 |
|  | BL - EOT | 1.246 | 0.146 - 10.665 | 0.841 |
| Weight loss | (Intercept) | 1.132 | 0.569 - 2.252 | 0.724 |
|  | BL - week 12 | 0.290 | 0.103 - 1.230 | 0.019 |
|  | BL - EOT | 0.344 | 0.121 - 0.980 | 0.046 |
| Weight gain | (Intercept) | 0.011 | 0.000 - 3.762 | 0.129 |
|  | BL - week 12 | 1.039 | 0.200 - 5.382 | 0.964 |
|  | BL - EOT | 3.025 | 0.600 - 15.256 | 0.18 |

**Supplementary Table 5:** Summary: Mixed Models for each EORTC H&N 35 score by timepoints of interest (baseline (BL), at first CT assessment scheduled at week 12 (median time to assessment 13.0 weeks) and end of treatment (EOT). The timepoints are used as fixed effects and random intercept for each patient.

**Supplementary Figure 1**


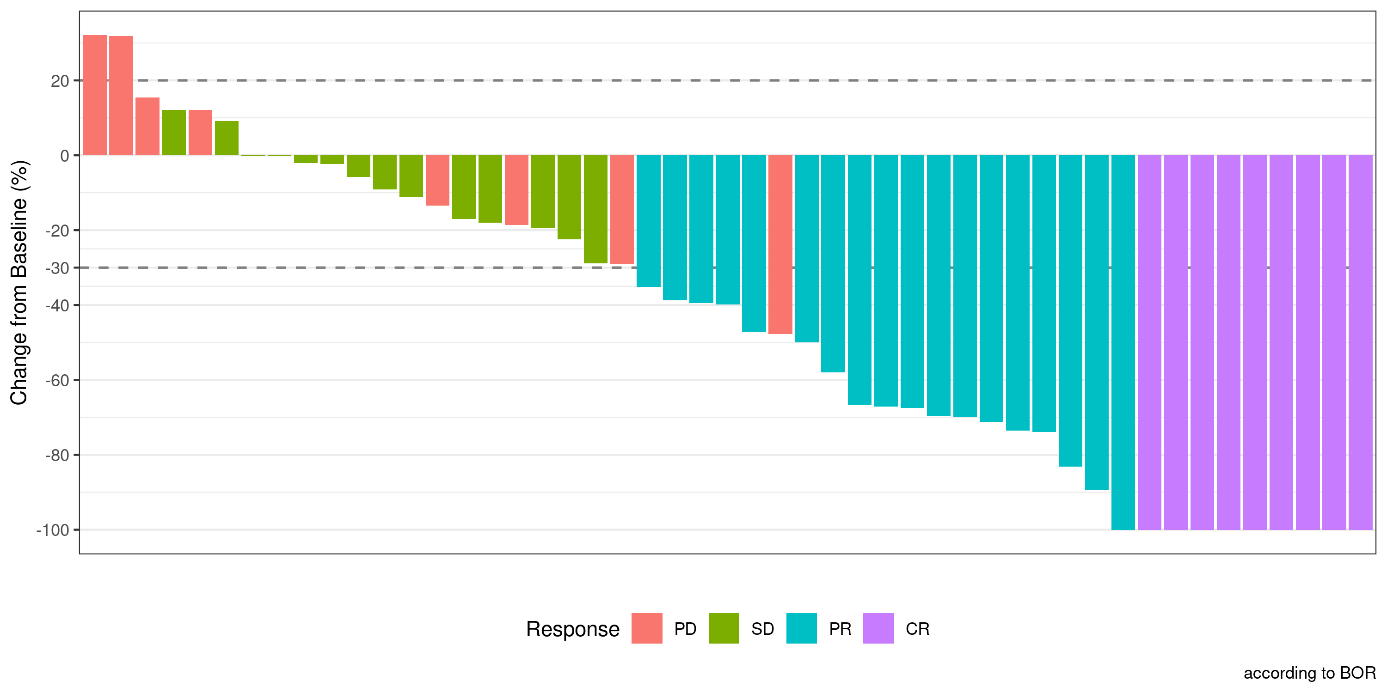


**Best overall response**

Waterfall plot illustrating best individual patients' responses. Complete Response (CR), partial response (PR), stable disease (SD) and progressive disease (PD).

**Supplementary Figure 2**

A


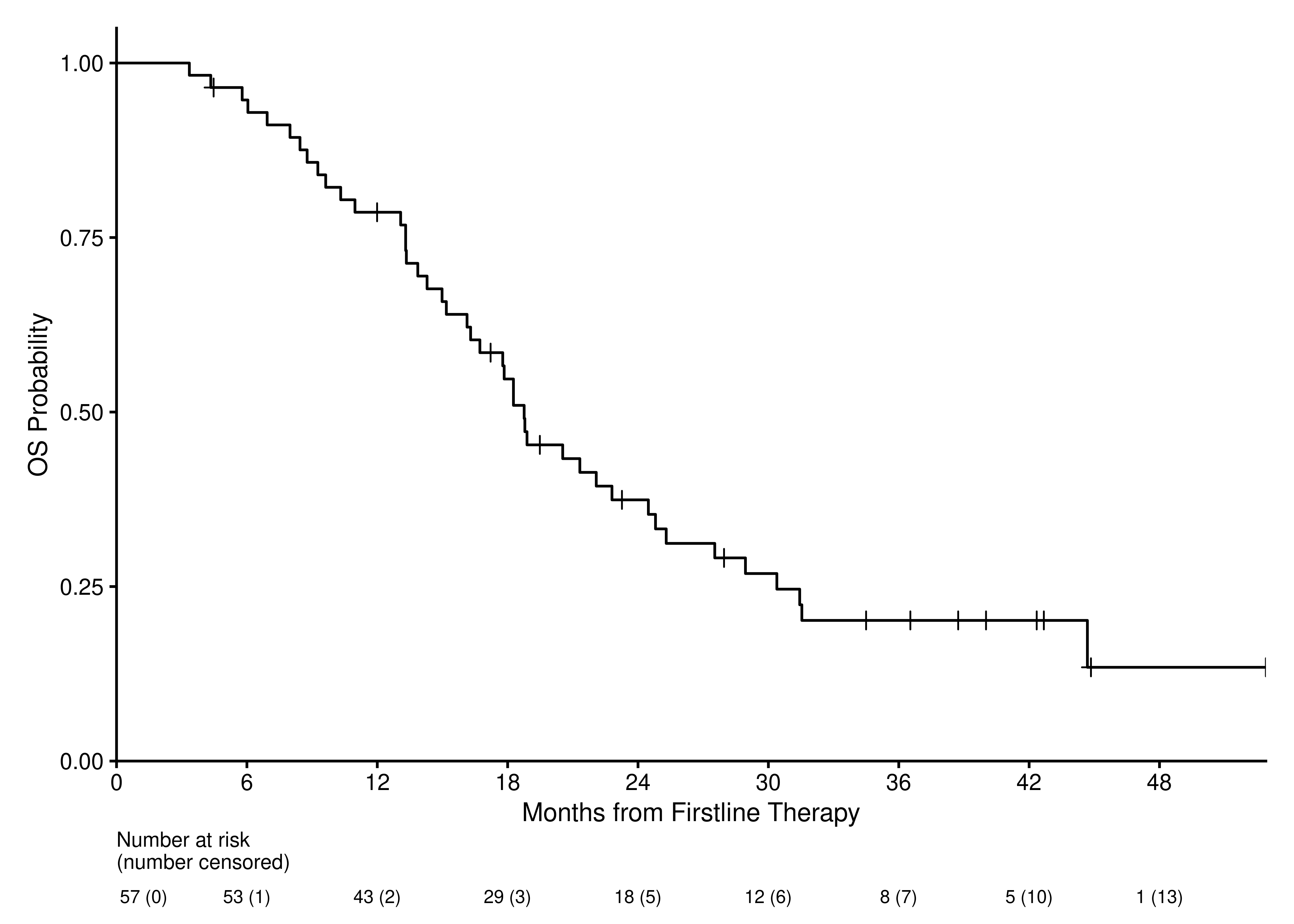


B


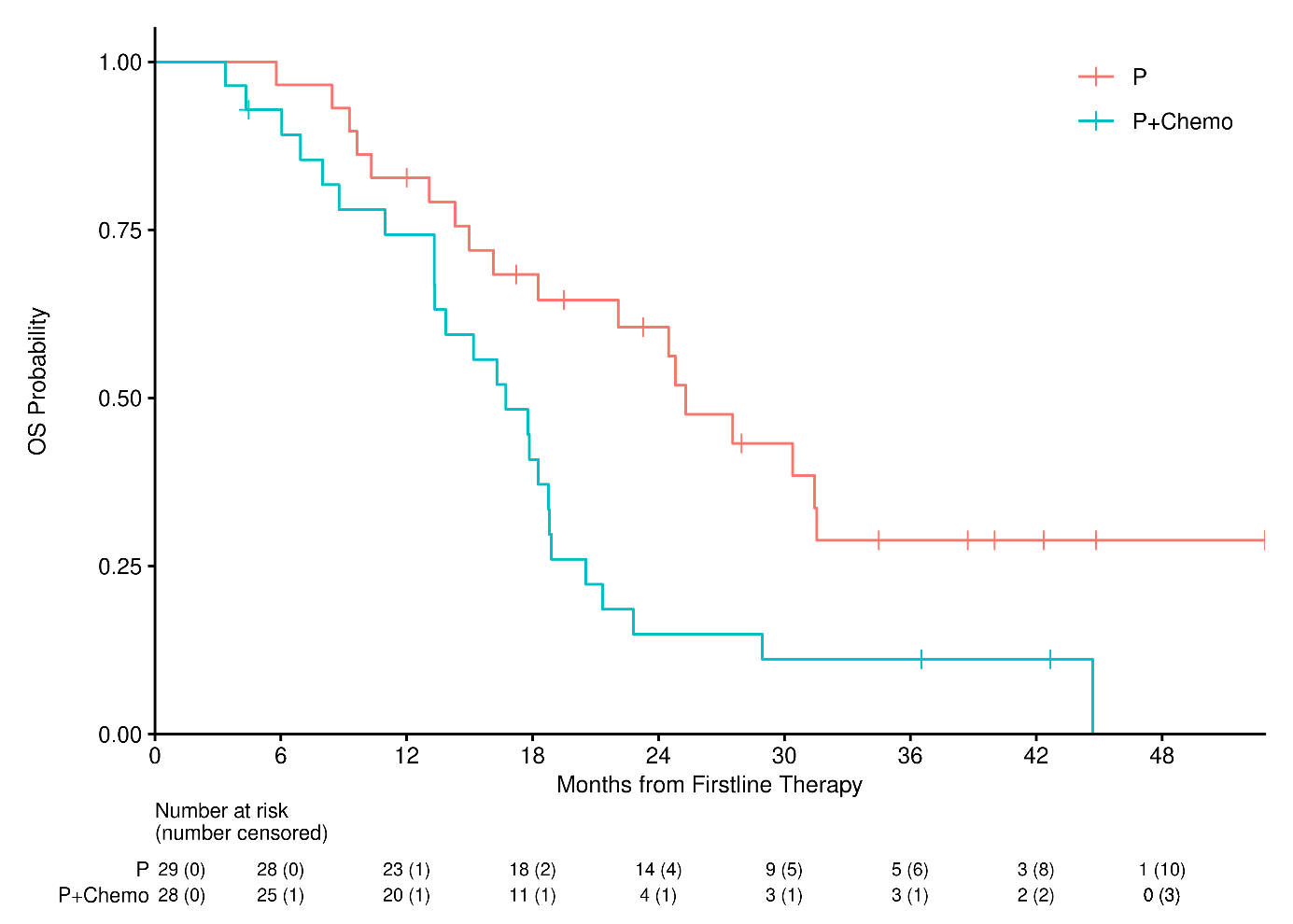


C


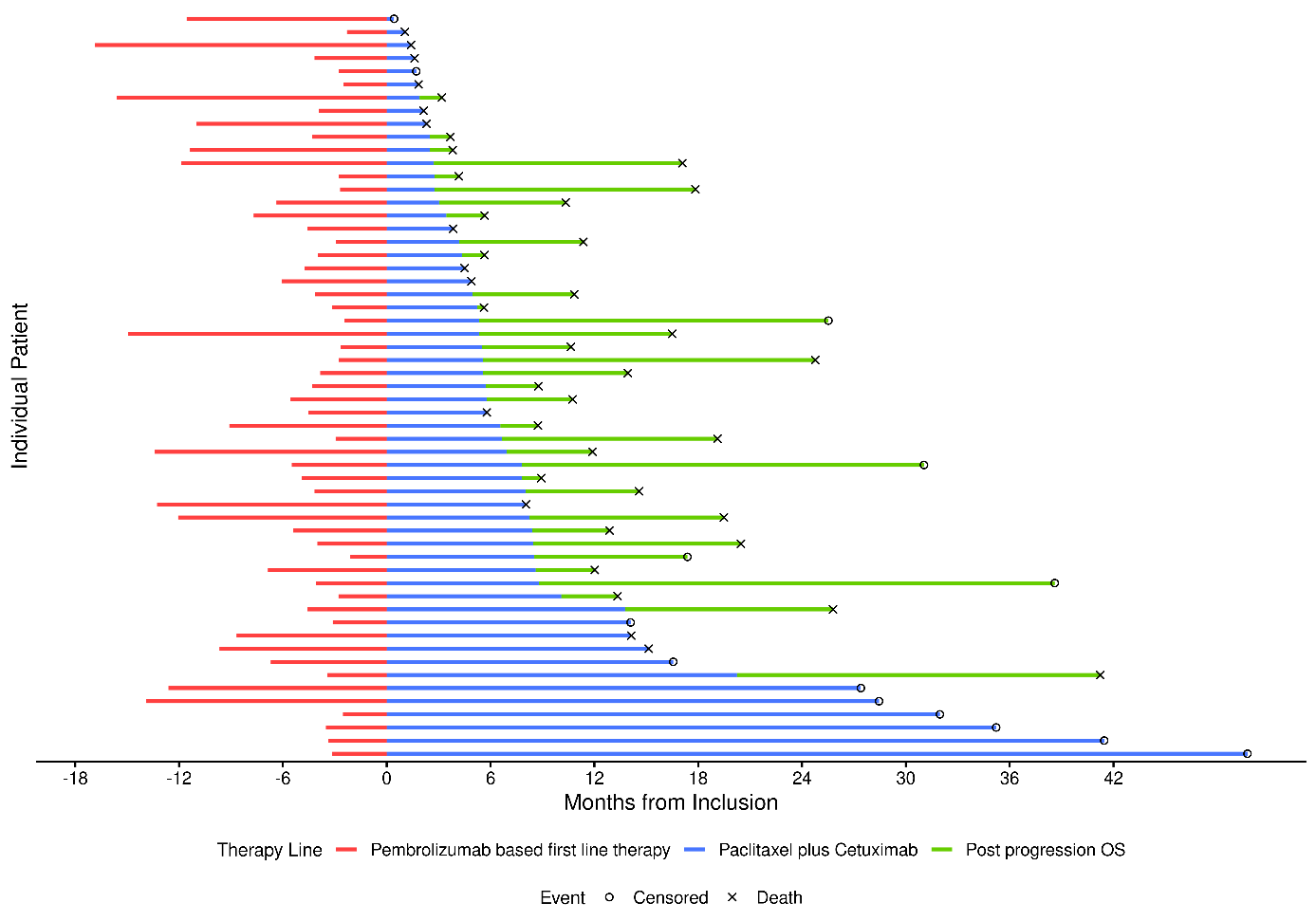


**Overall survival from first-line therapy and individual treatment courses.**

Kaplan-Meier curves depicting combined overall survival calculated from the start of first-line first line pembrolizumab therapy of the total population (A) and patients pre-treated with pembrolizumab (P) monotherapy (red) or pembrolizumab plus chemotherapy (P+Chemo) (turquoise) (B). Individual treatment course per therapy (C) line for first line immunotherapy (red), during PaceAce (blue) and post-progression (green). Circles indicate censored patients and crosses indicate patient’s death.
